# (*E*)-1-(6-Chloro-2-methyl-4-phenyl-3-quinol­yl)-3-(4-ethoxy­phen­yl)prop-2-en-1-one

**DOI:** 10.1107/S1600536810001248

**Published:** 2010-01-16

**Authors:** Tara Shahani, Hoong-Kun Fun, S. Sarveswari, V. Vijayakumar, R.Venkat Ragavan

**Affiliations:** a X-ray Crystallography Unit, School of Physics, Universiti Sains Malaysia, 11800 USM, Penang, Malaysia; b Organic Chemistry Division, School of Advanced Sciences, VIT University, Vellore 632 014, India

## Abstract

In the title compound, C_27_H_22_ClNO_2_, the phenyl substituent on the quinoline ring system is almost perpendicular to it [dihedral angle = 88.2 (1)°]. The quinoline ring system and the ethoxy­phenyl ring are oriented at dihedral angles of 79.5 (1) and 17.6 (3)°, respectively, with respect to the almost planar [r.m.s. deviation= 0.037 (3) Å] –C(=O)—C=C– linkage. In the crystal, the inversion-related mol­ecules exist as C—H⋯O hydrogen-bonded *R*_2_^2^(8) dimers.

## Related literature

For the biological activity of chalcone derivatives, see: Dimmock *et al.* (1999 ▶); Zi & Simoneau (2005 ▶); Yamazaki *et al.* (2002 ▶). For a related structure, see: Wu *et al.* (2006 ▶). For hydrogen-bond motifs, see: Bernstein *et al.* (1995 ▶). For bond-length data, see: Allen *et al.* (1987 ▶).
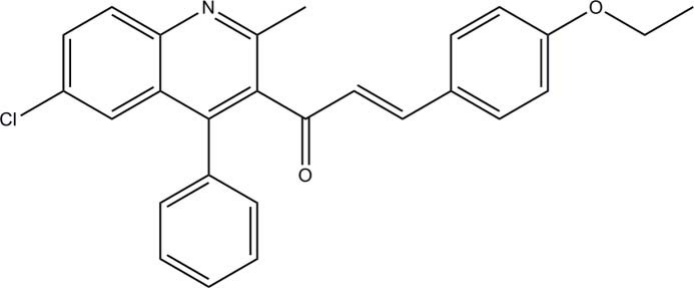


## Experimental

### 

#### Crystal data

C_27_H_22_ClNO_2_
*M* *_r_* = 427.91
Monoclinic, 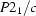

*a* = 16.2086 (5) Å
*b* = 13.4760 (4) Å
*c* = 10.5450 (3) Å
β = 105.128 (2)°
*V* = 2223.49 (11) Å^3^
*Z* = 4
Mo *K*α radiation
μ = 0.20 mm^−1^
*T* = 296 K
0.52 × 0.14 × 0.07 mm

#### Data collection

Bruker SMART APEXII CCD area-detector diffractometer
Absorption correction: multi-scan (*SADABS*; Bruker, 2009 ▶) *T*_min_ = 0.905, *T*_max_ = 0.986
39732 measured reflections
6511 independent reflections
2360 reflections with *I* > 2σ(*I*)
*R*_int_ = 0.110

#### Refinement

*R*[*F* ^2^ > 2σ(*F* ^2^)] = 0.068
*wR*(*F* ^2^) = 0.169
*S* = 1.00
6511 reflections
282 parameters
H-atom parameters constrained
Δρ_max_ = 0.16 e Å^−3^
Δρ_min_ = −0.16 e Å^−3^

### 

Data collection: *APEX2* (Bruker, 2009 ▶); cell refinement: *SAINT* (Bruker, 2009 ▶); data reduction: *SAINT*; program(s) used to solve structure: *SHELXTL* (Sheldrick, 2008 ▶); program(s) used to refine structure: *SHELXTL*; molecular graphics: *SHELXTL*; software used to prepare material for publication: *SHELXTL* and *PLATON* (Spek, 2009 ▶).

## Supplementary Material

Crystal structure: contains datablocks global, I. DOI: 10.1107/S1600536810001248/ci5014sup1.cif

Structure factors: contains datablocks I. DOI: 10.1107/S1600536810001248/ci5014Isup2.hkl

Additional supplementary materials:  crystallographic information; 3D view; checkCIF report

## Figures and Tables

**Table 1:** Hydrogen-bond geometry (Å, °)

*D*—H⋯*A*	*D*—H	H⋯*A*	*D*⋯*A*	*D*—H⋯*A*
C21—H21*A*⋯O2^i^	0.93	2.57	3.493 (3)	172

Symmetry code: (i) 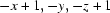.

**Fig. 1.**
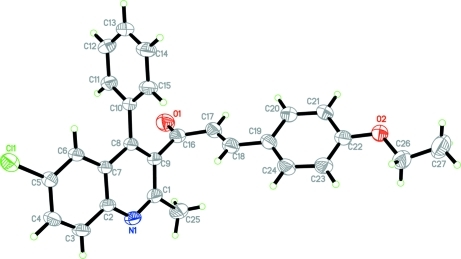
The molecular structure of the title compound, showing 30% probability displacement ellipsoids and the atom numbering scheme.

**Fig. 2.**
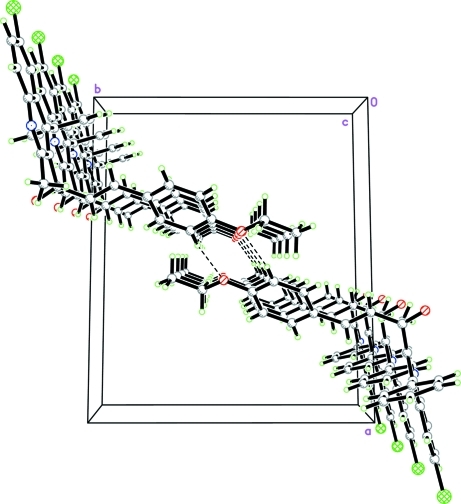
Part of the crystal packing of the title compound, viewed along the c axis.

## supplementary crystallographic information

### Comment

Chalcones are open chain flavonoids possessing a variety of biological activities such as antioxidant, anti-inflammation, antimicrobial, antiprotozoal, antiulcer, as well as other activities (Dimmock *et al.*, 1999). More importantly, chalcones have shown several anticancer activities as inhibitors of cancer cell proliferation, carcinogenesis and metastasis (Zi & Simoneau, 2005; Yamazaki *et al.*, 2002). We report here the crystal structure of the title chalcone derivative.

In the title molecule (Fig. 1), the quinoline ring system (C1/N1/C2–C9) is essentially planar with a maximum deviation of 0.026 (2) Å for atom C2. The C10–C15 and C19–C24 rings form dihedral angles of 88.2 (1)° and 67.8 (1)°, respectively, with the quinoline ring system. The ethoxy group is almost coplanar with the attached ring [C26—O2—C22—C23 = 1.8 (4)° and C22—O2—C26—C27 = -171.7 (3)°]. Bond lengths (Allen *et al.*, 1987) and angles show normal values.

In the crystal packing (Fig. 2), pairs of intermolecular C21—H21A···O2 hydrogen bonds (Table 1) form dimers with neighbouring molecules, generating *R*^2^_2_(8) ring motifs (Bernstein *et al.*, 1995). The dimers are stacked down the *c* axis (Fig. 2).

### Experimental

A mixture of 3-acetyl-6-chloro-2-methyl-4-phenylquinoline (2.95 g, 0.01 mmol), 4-ethoxybenzaldehyde (1.50 g, 0.01 mmol) and a catalytic amount of KOH in distilled ethanol was stirred for 12 h. The resulting mixture was concentrated to remove the ethanol and then poured onto ice and neutralized with diluted acetic acid. The resultant solid was filtered, dried and purified by column chromatography using a 1:1 mixture of ethylacetate and petroleum ether (m.p 401–403 K).

### Refinement

H atoms were positioned geometrically [C–H = 0.93–0.97 Å] and refined using a riding model, with *U*_iso_(H) = 1.2-1.5*U*_eq_(C). A rotating group model was used for the methyl groups. The ratio of observed to unique reflections is low (36%), and the value of R_int_ is greater than 0.10, probably due to the poor diffraction quality of the crystal.

### Crystal data

**Table:**

C_27_H_22_ClNO_2_	*F*(000) = 896
*M**_r_* = 427.91	*D*_x_ = 1.278 Mg m^−^^3^
Monoclinic, *P*2_1_/*c*	Mo *K*α radiation, λ = 0.71073 Å
Hall symbol: -P 2ybc	Cell parameters from 2856 reflections
*a* = 16.2086 (5) Å	θ = 2.5–19.7°
*b* = 13.4760 (4) Å	µ = 0.20 mm^−^^1^
*c* = 10.5450 (3) Å	*T* = 296 K
β = 105.128 (2)°	Plate, colourless
*V* = 2223.49 (11) Å^3^	0.52 × 0.14 × 0.07 mm
*Z* = 4	

### Data collection

**Table:**

Bruker SMART APEXII CCD area-detector diffractometer	6511 independent reflections
Radiation source: fine-focus sealed tube	2360 reflections with *I* > 2σ(*I*)
graphite	*R*_int_ = 0.110
φ and ω scans	θ_max_ = 30.1°, θ_min_ = 2.0°
Absorption correction: multi-scan (*SADABS*; Bruker, 2009)	*h* = −22→22
*T*_min_ = 0.905, *T*_max_ = 0.986	*k* = −18→19
39732 measured reflections	*l* = −14→14

### Refinement

**Table:**

Refinement on *F*^2^	Primary atom site location: structure-invariant direct methods
Least-squares matrix: full	Secondary atom site location: difference Fourier map
*R*[*F*^2^ > 2σ(*F*^2^)] = 0.068	Hydrogen site location: inferred from neighbouring sites
*wR*(*F*^2^) = 0.169	H-atom parameters constrained
*S* = 1.00	*w* = 1/[σ^2^(*F*_o_^2^) + (0.0605*P*)^2^] where *P* = (*F*_o_^2^ + 2*F*_c_^2^)/3
6511 reflections	(Δ/σ)_max_ = 0.001
282 parameters	Δρ_max_ = 0.16 e Å^−^^3^
0 restraints	Δρ_min_ = −0.16 e Å^−^^3^

### Special details

**Table:**

Experimental. The crystal was placed in the cold stream of an Oxford Cyrosystems Cobra open-flow nitrogen cryostat (Cosier & Glazer, 1986) operating at 100.0 (1) K.
Geometry. All e.s.d.'s (except the e.s.d. in the dihedral angle between two l.s. planes) are estimated using the full covariance matrix. The cell e.s.d.'s are taken into account individually in the estimation of e.s.d.'s in distances, angles and torsion angles; correlations between e.s.d.'s in cell parameters are only used when they are defined by crystal symmetry. An approximate (isotropic) treatment of cell e.s.d.'s is used for estimating e.s.d.'s involving l.s. planes.
Refinement. Refinement of *F*^2^ against ALL reflections. The weighted *R*-factor *wR* and goodness of fit *S* are based on *F*^2^, conventional *R*-factors *R* are based on *F*, with *F* set to zero for negative *F*^2^. The threshold expression of *F*^2^ > σ(*F*^2^) is used only for calculating *R*-factors(gt) *etc*. and is not relevant to the choice of reflections for refinement. *R*-factors based on *F*^2^ are statistically about twice as large as those based on *F*, and *R*- factors based on ALL data will be even larger.

### Fractional atomic coordinates and isotropic or equivalent isotropic displacement parameters (Å^2^)

**Table:**

	*x*	*y*	*z*	*U*_iso_*/*U*_eq_	
Cl1	1.16190 (5)	0.67801 (6)	0.88581 (7)	0.0828 (3)	
O1	0.64844 (13)	0.61168 (16)	0.6171 (2)	0.0913 (7)	
O2	0.56628 (11)	−0.02725 (14)	0.68435 (17)	0.0742 (5)	
N1	0.83573 (15)	0.60187 (15)	1.01410 (19)	0.0652 (6)	
C1	0.76675 (18)	0.57733 (18)	0.9210 (3)	0.0636 (7)	
C2	0.91019 (17)	0.61647 (17)	0.9804 (2)	0.0561 (6)	
C3	0.98340 (19)	0.64653 (18)	1.0782 (2)	0.0681 (8)	
H3A	0.9796	0.6536	1.1643	0.082*	
C4	1.05818 (18)	0.66517 (19)	1.0508 (2)	0.0669 (8)	
H4A	1.1052	0.6855	1.1169	0.080*	
C5	1.06500 (16)	0.65382 (17)	0.9211 (2)	0.0598 (7)	
C6	0.99656 (16)	0.62385 (17)	0.8232 (2)	0.0571 (7)	
H6A	1.0023	0.6157	0.7383	0.068*	
C7	0.91777 (16)	0.60528 (16)	0.8499 (2)	0.0516 (6)	
C8	0.84261 (17)	0.57688 (16)	0.7512 (2)	0.0533 (6)	
C9	0.76818 (16)	0.56417 (18)	0.7873 (2)	0.0568 (6)	
C10	0.84861 (15)	0.56255 (19)	0.6129 (2)	0.0535 (6)	
C11	0.83198 (18)	0.6399 (2)	0.5248 (3)	0.0722 (8)	
H11A	0.8149	0.7011	0.5499	0.087*	
C12	0.8408 (2)	0.6264 (3)	0.3979 (3)	0.0832 (9)	
H12A	0.8296	0.6789	0.3386	0.100*	
C13	0.86553 (19)	0.5372 (3)	0.3599 (3)	0.0793 (9)	
H13A	0.8711	0.5287	0.2751	0.095*	
C14	0.88206 (19)	0.4605 (2)	0.4470 (3)	0.0809 (9)	
H14A	0.8990	0.3994	0.4213	0.097*	
C15	0.87381 (17)	0.4728 (2)	0.5731 (2)	0.0696 (8)	
H15A	0.8854	0.4200	0.6318	0.084*	
C16	0.68610 (18)	0.5431 (2)	0.6824 (3)	0.0674 (7)	
C17	0.65339 (17)	0.4419 (2)	0.6605 (3)	0.0714 (8)	
H17A	0.6050	0.4311	0.5917	0.086*	
C18	0.68782 (17)	0.3647 (2)	0.7317 (3)	0.0701 (8)	
H18A	0.7369	0.3770	0.7985	0.084*	
C19	0.65760 (16)	0.2617 (2)	0.7177 (3)	0.0647 (7)	
C20	0.59824 (17)	0.2271 (2)	0.6052 (2)	0.0719 (8)	
H20A	0.5783	0.2696	0.5344	0.086*	
C21	0.56885 (17)	0.1309 (2)	0.5978 (3)	0.0703 (8)	
H21A	0.5291	0.1092	0.5224	0.084*	
C22	0.59809 (16)	0.0660 (2)	0.7018 (2)	0.0630 (7)	
C23	0.65787 (18)	0.0995 (2)	0.8127 (3)	0.0750 (8)	
H23A	0.6782	0.0570	0.8835	0.090*	
C24	0.68682 (17)	0.1947 (2)	0.8180 (3)	0.0747 (8)	
H24A	0.7280	0.2153	0.8923	0.090*	
C25	0.68576 (19)	0.5650 (2)	0.9637 (3)	0.0906 (10)	
H25A	0.6887	0.6049	1.0401	0.136*	
H25B	0.6790	0.4965	0.9842	0.136*	
H25C	0.6379	0.5855	0.8940	0.136*	
C26	0.5940 (2)	−0.0967 (2)	0.7886 (3)	0.0938 (10)	
H26A	0.5725	−0.0777	0.8627	0.113*	
H26B	0.6560	−0.0977	0.8171	0.113*	
C27	0.5619 (2)	−0.1949 (3)	0.7416 (4)	0.1204 (13)	
H27A	0.5787	−0.2420	0.8119	0.181*	
H27B	0.5853	−0.2142	0.6705	0.181*	
H27C	0.5007	−0.1930	0.7115	0.181*	

### Atomic displacement parameters (Å^2^)

**Table:**

	*U*^11^	*U*^22^	*U*^33^	*U*^12^	*U*^13^	*U*^23^
Cl1	0.0758 (5)	0.1044 (6)	0.0655 (5)	−0.0208 (4)	0.0137 (4)	−0.0139 (4)
O1	0.0965 (16)	0.0922 (16)	0.0772 (14)	0.0004 (12)	0.0081 (12)	0.0054 (12)
O2	0.0882 (13)	0.0688 (13)	0.0590 (11)	−0.0121 (10)	0.0075 (10)	0.0023 (10)
N1	0.0898 (17)	0.0651 (15)	0.0463 (12)	−0.0059 (12)	0.0280 (12)	−0.0022 (11)
C1	0.0804 (19)	0.0612 (18)	0.0541 (16)	−0.0052 (14)	0.0260 (15)	−0.0026 (13)
C2	0.0806 (18)	0.0504 (15)	0.0389 (13)	−0.0023 (13)	0.0184 (13)	−0.0007 (11)
C3	0.094 (2)	0.0725 (19)	0.0371 (14)	−0.0044 (16)	0.0162 (14)	−0.0061 (12)
C4	0.081 (2)	0.0707 (19)	0.0420 (15)	−0.0070 (15)	0.0034 (13)	−0.0071 (12)
C5	0.0687 (16)	0.0581 (16)	0.0504 (15)	−0.0073 (13)	0.0117 (13)	−0.0033 (12)
C6	0.0786 (18)	0.0543 (16)	0.0388 (13)	−0.0073 (13)	0.0163 (13)	−0.0064 (11)
C7	0.0695 (17)	0.0473 (14)	0.0390 (13)	−0.0068 (12)	0.0156 (12)	−0.0027 (11)
C8	0.0747 (17)	0.0473 (15)	0.0399 (13)	−0.0062 (12)	0.0183 (12)	−0.0037 (11)
C9	0.0734 (17)	0.0563 (16)	0.0447 (14)	−0.0080 (13)	0.0225 (13)	−0.0049 (12)
C10	0.0654 (15)	0.0565 (16)	0.0392 (13)	−0.0126 (13)	0.0149 (11)	−0.0048 (12)
C11	0.103 (2)	0.0622 (18)	0.0529 (16)	−0.0086 (15)	0.0231 (15)	0.0024 (14)
C12	0.114 (3)	0.087 (2)	0.0502 (17)	−0.0144 (19)	0.0245 (16)	0.0127 (16)
C13	0.098 (2)	0.097 (2)	0.0477 (16)	−0.0237 (19)	0.0279 (15)	−0.0128 (17)
C14	0.110 (2)	0.082 (2)	0.0560 (17)	−0.0013 (18)	0.0304 (16)	−0.0156 (16)
C15	0.098 (2)	0.0667 (19)	0.0445 (15)	0.0004 (16)	0.0189 (14)	−0.0033 (13)
C16	0.0715 (18)	0.079 (2)	0.0556 (17)	−0.0056 (16)	0.0246 (14)	−0.0067 (15)
C17	0.0646 (17)	0.086 (2)	0.0619 (17)	−0.0129 (16)	0.0134 (14)	−0.0095 (16)
C18	0.0642 (17)	0.087 (2)	0.0605 (17)	−0.0086 (16)	0.0194 (14)	−0.0137 (16)
C19	0.0628 (16)	0.074 (2)	0.0585 (17)	−0.0095 (14)	0.0173 (13)	−0.0157 (15)
C20	0.0791 (19)	0.077 (2)	0.0561 (16)	−0.0072 (16)	0.0119 (14)	−0.0001 (14)
C21	0.0769 (18)	0.075 (2)	0.0530 (16)	−0.0084 (15)	0.0059 (14)	−0.0069 (15)
C22	0.0636 (16)	0.073 (2)	0.0526 (16)	−0.0015 (14)	0.0158 (13)	−0.0058 (14)
C23	0.0748 (19)	0.088 (2)	0.0554 (17)	−0.0078 (16)	0.0048 (14)	0.0013 (15)
C24	0.0687 (18)	0.088 (2)	0.0593 (17)	−0.0090 (16)	0.0017 (14)	−0.0070 (17)
C25	0.097 (2)	0.116 (3)	0.0730 (19)	−0.0114 (19)	0.0472 (17)	−0.0061 (18)
C26	0.103 (2)	0.094 (3)	0.073 (2)	−0.0185 (19)	0.0026 (18)	0.0186 (19)
C27	0.141 (3)	0.099 (3)	0.119 (3)	−0.012 (2)	0.029 (2)	0.034 (2)

### Geometric parameters (Å, °)

**Table:**

Cl1—C5	1.737 (3)	C13—H13A	0.93
O1—C16	1.217 (3)	C14—C15	1.382 (3)
O2—C22	1.352 (3)	C14—H14A	0.93
O2—C26	1.424 (3)	C15—H15A	0.93
N1—C1	1.323 (3)	C16—C17	1.460 (4)
N1—C2	1.359 (3)	C17—C18	1.319 (4)
C1—C9	1.427 (3)	C17—H17A	0.93
C1—C25	1.504 (4)	C18—C19	1.467 (4)
C2—C3	1.414 (3)	C18—H18A	0.93
C2—C7	1.421 (3)	C19—C24	1.376 (4)
C3—C4	1.341 (3)	C19—C20	1.398 (3)
C3—H3A	0.93	C20—C21	1.376 (4)
C4—C5	1.408 (3)	C20—H20A	0.93
C4—H4A	0.93	C21—C22	1.386 (3)
C5—C6	1.365 (3)	C21—H21A	0.93
C6—C7	1.400 (3)	C22—C23	1.385 (3)
C6—H6A	0.93	C23—C24	1.362 (4)
C7—C8	1.432 (3)	C23—H23A	0.93
C8—C9	1.367 (3)	C24—H24A	0.93
C8—C10	1.500 (3)	C25—H25A	0.96
C9—C16	1.518 (4)	C25—H25B	0.96
C10—C11	1.375 (3)	C25—H25C	0.96
C10—C15	1.376 (3)	C26—C27	1.460 (4)
C11—C12	1.395 (4)	C26—H26A	0.97
C11—H11A	0.93	C26—H26B	0.97
C12—C13	1.360 (4)	C27—H27A	0.96
C12—H12A	0.93	C27—H27B	0.96
C13—C14	1.362 (4)	C27—H27C	0.96
			
C22—O2—C26	118.2 (2)	C14—C15—H15A	119.7
C1—N1—C2	118.6 (2)	O1—C16—C17	120.8 (3)
N1—C1—C9	122.4 (2)	O1—C16—C9	119.0 (3)
N1—C1—C25	116.1 (2)	C17—C16—C9	120.2 (3)
C9—C1—C25	121.5 (2)	C18—C17—C16	124.4 (3)
N1—C2—C3	119.0 (2)	C18—C17—H17A	117.8
N1—C2—C7	123.1 (2)	C16—C17—H17A	117.8
C3—C2—C7	118.0 (3)	C17—C18—C19	127.5 (3)
C4—C3—C2	122.0 (2)	C17—C18—H18A	116.2
C4—C3—H3A	119.0	C19—C18—H18A	116.2
C2—C3—H3A	119.0	C24—C19—C20	117.1 (3)
C3—C4—C5	119.6 (2)	C24—C19—C18	120.3 (2)
C3—C4—H4A	120.2	C20—C19—C18	122.6 (3)
C5—C4—H4A	120.2	C21—C20—C19	120.9 (3)
C6—C5—C4	120.8 (3)	C21—C20—H20A	119.6
C6—C5—Cl1	119.7 (2)	C19—C20—H20A	119.6
C4—C5—Cl1	119.5 (2)	C20—C21—C22	120.5 (2)
C5—C6—C7	120.3 (2)	C20—C21—H21A	119.7
C5—C6—H6A	119.8	C22—C21—H21A	119.7
C7—C6—H6A	119.8	O2—C22—C23	125.1 (3)
C6—C7—C2	119.3 (2)	O2—C22—C21	116.0 (2)
C6—C7—C8	123.3 (2)	C23—C22—C21	118.8 (3)
C2—C7—C8	117.4 (2)	C24—C23—C22	119.9 (3)
C9—C8—C7	118.5 (2)	C24—C23—H23A	120.0
C9—C8—C10	122.7 (2)	C22—C23—H23A	120.0
C7—C8—C10	118.8 (2)	C23—C24—C19	122.7 (2)
C8—C9—C1	120.1 (2)	C23—C24—H24A	118.6
C8—C9—C16	119.3 (2)	C19—C24—H24A	118.6
C1—C9—C16	120.4 (2)	C1—C25—H25A	109.5
C11—C10—C15	118.8 (2)	C1—C25—H25B	109.5
C11—C10—C8	120.5 (2)	H25A—C25—H25B	109.5
C15—C10—C8	120.6 (2)	C1—C25—H25C	109.5
C10—C11—C12	119.9 (3)	H25A—C25—H25C	109.5
C10—C11—H11A	120.1	H25B—C25—H25C	109.5
C12—C11—H11A	120.1	O2—C26—C27	108.7 (2)
C13—C12—C11	120.6 (3)	O2—C26—H26A	109.9
C13—C12—H12A	119.7	C27—C26—H26A	109.9
C11—C12—H12A	119.7	O2—C26—H26B	109.9
C12—C13—C14	119.6 (3)	C27—C26—H26B	109.9
C12—C13—H13A	120.2	H26A—C26—H26B	108.3
C14—C13—H13A	120.2	C26—C27—H27A	109.5
C13—C14—C15	120.4 (3)	C26—C27—H27B	109.5
C13—C14—H14A	119.8	H27A—C27—H27B	109.5
C15—C14—H14A	119.8	C26—C27—H27C	109.5
C10—C15—C14	120.7 (3)	H27A—C27—H27C	109.5
C10—C15—H15A	119.7	H27B—C27—H27C	109.5
			
C2—N1—C1—C9	1.0 (4)	C7—C8—C10—C15	86.0 (3)
C2—N1—C1—C25	−178.4 (2)	C15—C10—C11—C12	0.0 (4)
C1—N1—C2—C3	177.5 (2)	C8—C10—C11—C12	177.7 (3)
C1—N1—C2—C7	−0.9 (4)	C10—C11—C12—C13	0.2 (4)
N1—C2—C3—C4	−177.9 (2)	C11—C12—C13—C14	−0.2 (5)
C7—C2—C3—C4	0.6 (4)	C12—C13—C14—C15	0.0 (5)
C2—C3—C4—C5	−0.6 (4)	C11—C10—C15—C14	−0.2 (4)
C3—C4—C5—C6	−0.2 (4)	C8—C10—C15—C14	−177.9 (3)
C3—C4—C5—Cl1	180.0 (2)	C13—C14—C15—C10	0.2 (4)
C4—C5—C6—C7	1.0 (4)	C8—C9—C16—O1	−79.3 (3)
Cl1—C5—C6—C7	−179.20 (18)	C1—C9—C16—O1	96.4 (3)
C5—C6—C7—C2	−1.0 (4)	C8—C9—C16—C17	100.5 (3)
C5—C6—C7—C8	177.8 (2)	C1—C9—C16—C17	−83.8 (3)
N1—C2—C7—C6	178.6 (2)	O1—C16—C17—C18	−176.1 (3)
C3—C2—C7—C6	0.2 (3)	C9—C16—C17—C18	4.1 (4)
N1—C2—C7—C8	−0.3 (3)	C16—C17—C18—C19	178.4 (2)
C3—C2—C7—C8	−178.7 (2)	C17—C18—C19—C24	−163.6 (3)
C6—C7—C8—C9	−177.6 (2)	C17—C18—C19—C20	15.7 (4)
C2—C7—C8—C9	1.3 (3)	C24—C19—C20—C21	1.9 (4)
C6—C7—C8—C10	2.3 (3)	C18—C19—C20—C21	−177.4 (3)
C2—C7—C8—C10	−178.9 (2)	C19—C20—C21—C22	−0.4 (4)
C7—C8—C9—C1	−1.2 (3)	C26—O2—C22—C23	1.8 (4)
C10—C8—C9—C1	179.0 (2)	C26—O2—C22—C21	−179.7 (3)
C7—C8—C9—C16	174.6 (2)	C20—C21—C22—O2	−179.1 (3)
C10—C8—C9—C16	−5.2 (4)	C20—C21—C22—C23	−0.5 (4)
N1—C1—C9—C8	0.0 (4)	O2—C22—C23—C24	178.4 (3)
C25—C1—C9—C8	179.4 (2)	C21—C22—C23—C24	0.0 (4)
N1—C1—C9—C16	−175.7 (2)	C22—C23—C24—C19	1.6 (4)
C25—C1—C9—C16	3.7 (4)	C20—C19—C24—C23	−2.5 (4)
C9—C8—C10—C11	88.1 (3)	C18—C19—C24—C23	176.8 (3)
C7—C8—C10—C11	−91.7 (3)	C22—O2—C26—C27	−171.7 (3)
C9—C8—C10—C15	−94.2 (3)		

### Hydrogen-bond geometry (Å, °)

**Table:**

*D*—H···*A*	*D*—H	H···*A*	*D*···*A*	*D*—H···*A*
C21—H21A···O2^i^	0.93	2.57	3.493 (3)	172

Symmetry codes: (i) −*x*+1, −*y*, −*z*+1.
